# Differences Between Younger and Older US Adults With Multiple Chronic Conditions

**DOI:** 10.5888/pcd14.160613

**Published:** 2017-09-07

**Authors:** Mary L. Adams

## Abstract

**Introduction:**

Adults with multiple (≥2) chronic conditions (MCCs) account for a large portion of US health care costs. Despite the increase in MCC rates with age, most people with MCCs are working age. The study objective was to compare adults with MCCs who were younger than 65 years with those aged 65 years or older on selected measures to better understand the differences between groups and inform interventions that could lower health care costs.

**Methods:**

Data from respondents to the 2015 Behavioral Risk Factor Surveillance System data (N = 201,711) were used to compare adults aged 65 or older with MCCs with those younger than 65 with MCCs in unadjusted and adjusted analyses on chronic conditions, quality of life measures, disability status, access to health care, and modifiable risk factors. MCCs were based on up to 12 chronic conditions (heart disease, stroke, asthma, arthritis, chronic obstructive pulmonary disease, high cholesterol, cognitive impairment, diabetes, depression, chronic kidney disease, cancer other than skin, and hypertension).

**Results:**

Consistent with 80% of all adults being younger than 65, more than 60% of adults with MCCs were younger than 65 years. Compared with adults aged 65 or older with MCCs, those younger than 65 were more likely to report asthma, cognitive impairment, depression, smoking, obesity, poorer access to health care, disability, and worse quality of life in both unadjusted and adjusted analysis.

**Conclusion:**

To decrease the burden of chronic diseases, adults younger than 65 with MCCs should get the treatment they need to reduce the chance of developing more chronic conditions as they age. The ultimate goal is to improve health status and reduce health care costs for everyone with MCCs.

## Introduction

Rates of multiple chronic conditions (MCCs), defined as having 2 or more co-occurring chronic conditions, tend to increase with age (1), resulting in increasing Medicare costs (2). As a consequence, considerable information on MCCs comes from Medicare claims data (3,4) for adults aged 65 years or older. A recent review of 163 studies (5) that included adults of all ages noted that despite the increase in MCC rates with age, most people with MCCs are working age. Other studies found that medical expenditures for chronic conditions among nonelderly adults and adults aged 65 or older were similar, averaging approximately $3,700 for those with 2 or 3 chronic conditions and $8,900 for those with 4 or more (6). Another study (7) found larger relative increases in MCCs over time among those aged 25 to 44 years compared with older adults and different chronic conditions by age group.

Lifestyle factors such as smoking and obesity (8–10) increase the risk of many chronic conditions included in measures of MCCs. Some chronic conditions, such as diabetes, depression, high blood pressure, and high cholesterol, are also risk factors for MCCs (5). Any of these risk factors can increase the likelihood of developing additional chronic conditions in adults of any age. When considered collectively, these findings suggest the need for a better understanding of MCCs among younger adults to develop effective strategies to prevent more chronic conditions from developing and better manage existing ones. This understanding in turn could mitigate any increase in future health care costs.

The objective of this study was to compare adults younger than 65 with MCCs with those aged 65 years or older with MCCs on selected measures. Measures included disability status, quality of life measures, chronic conditions, risk factors, and access to health care to add to information that is known about adults younger than 65 with MCCs. Because there is no standard list of chronic conditions to include, the study used different definitions of MCCs: one that considers diabetes, high blood pressure, high cholesterol, and depression as chronic conditions, and one that does not.

## Methods

We examined data from the 2015 Behavioral Risk Factor Surveillance System (BRFSS) data (11) on 434,382 respondents aged 18 years or older in the 50 states and the District of Columbia. BRFSS data have been shown to be comparable to those of other surveys, with most self-reported measures showing good or better validity (12). For all measures used, responses of “don’t know” or refusal to answer were excluded. The median response rate for cellular telephone and landline surveys combined for the 50 states and the District of Columbia was 46.6%, ranging from 33.9% in California to 61.1% in Utah (13). Data were weighted to be representative of the total adult population of each state by age, race/ethnicity, sex, marital status, education, home ownership, and type of telephone service. Chronic conditions were chosen to be as consistent as possible with commonly cited lists (14,15) and availability on the BRFSS. The MCC based on 5 chronic conditions (MCC5) included cardiovascular disease (CVD; heart attack, angina, coronary heart disease, or stroke), asthma, chronic obstructive pulmonary disease (COPD), arthritis, and cognitive impairment while MCC12 split CVD into heart disease and stroke and added diabetes, high blood pressure, depression, high cholesterol, chronic kidney disease, and cancer other than skin. All chronic conditions except cognitive impairment were defined as having “ever been told”; women who were told they had diabetes only when pregnant were considered not to have diabetes. Cognitive impairment was defined as a yes response to “Because of a physical, mental, or emotional problem, do you have difficulty remembering, concentrating, or making decisions?” This question has been asked by the US Census Bureau since 2008 and is now asked as 1 of 6 disability questions on all federal surveys (16). This cognitive impairment measure is consistent with other measures of cognitive impairment but should not be considered cognitive decline, because the question lacks a time frame (17,18). Respondents who reported any 2 or more of the 5 chronic conditions were considered to have MCC5 (n = 69,487), and those who reported any 2 or more of the 12 were considered to have MCC12 (n = 201,711).

Demographic measures were sex, age (18–64 y and ≥65 y), race/ethnicity (non-Hispanic white, black or African American, Hispanic of any race, American Indian/Alaska Native, and other), education level (college graduate, some college, high school graduate, <high school graduate), annual household income (≥$75,000, $50,000–$74,999, $25,000–$49,999, $15,000–24,999, <$15,000, or unknown), and employment status (employed/self-employed, out of work, homemaker, student, retired, or unable to work). The list of measures that were compared by age group included each of the chronic conditions included in the MCCs; disability (limited in any way in any activities because of physical, mental, or emotional problems); 4 difficulty measures (difficulty seeing, walking, bathing or dressing, or doing errands alone); obesity (body mass index ≥30 kg/m^2^ based on self-reported height and weight); current smoking; no leisure time physical activity (in the past month); no health insurance; no personal physician; a barrier to health care (needing to see a doctor in past year but unable to because of cost); having a routine check-up in the past 2 years; and quality of life measures including fair or poor general health, frequent (14 or more days in the past 30 days) poor mental health (referred to as frequent mental distress [FMD]), frequent poor physical health (FPD), and frequent activity limitation (FAL).

Stata version 14.1 (StataCorp LP) was used for all weighted data analysis to account for the complex sample design of the BRFSS, and Pearson *χ* ^2^ tests were used to determine significance at *P* <.05. Age group comparisons (<65 y vs ≥65 y) were made for adults with MCC12 and those without MCC12 and also for adults with MCC5. Logistic regression was conducted, controlling for demographic and other selected measures, to confirm unadjusted results.

## Results

The weighted percentage of all survey respondents younger than age 65 was 80.3% (Table 1). Although the percentage of adults with MCCs who were younger than age 65 was less than that for all adults, 61.4% of adults with MCC5 and 63.8% with MCC12 were younger than 65 years. Other demographic, health status, and insurance status characteristics varied between all adults and those with MCCs. Results of age group comparisons (Table 2) indicated that compared with adults aged 65 or older, younger adults were more likely to report a total of 18 measures including 10 that showed similar results for adults with and without MCC12 and 8 that only showed significantly worse results among adults ages 18 to 64 years with MCC12 (5 measures related to disability and 3 indicating poorer quality of life). Although adults with and without MCC12 were not directly compared, for all measures except health care access, adults with MCC12 in both age groups appeared worse off than those without MCC12 (Table 2). Results for measures among adults with MCC5 were similar but often higher for both age groups compared with results for MCC12 (Appendix).

**Table 1:** Demographic and Health Characteristics of Adults With Multiple Chronic Conditions, Behavioral Risk Factor Surveillance System, 2015

Characteristic	All (N = 434,382)	MCC12^a^ (N = 201,711)	MCC5^b^ (N = 69,487)
	Weighted %		
**Age, y**			
18–64	80.3	63.8	61.4
≥65	19.7	36.2	38.6
**Sex**			
Male	48.7	45.6	40.6
Female	51.3	54.4	59.4
**Race/ethnicity**			
White, non-Hispanic	64.4	71.1	72.2
Black, non-Hispanic	11.9	12.3	12.7
Hispanic any race	15.7	10.8	9.3
American Indian/Alaska Native	1.0	1.2	1.8
Other	7.0	4.7	4.0
**Education level**			
<High school graduate	14.2	16.5	22.8
High school graduate	28.3	29.9	31.8
Some college	31.1	31.6	31.5
College graduate	26.4	22.0	13.9
**Employment status**			
Employed or self-employed	56.9	40.4	24.9
Out of work	5.9	5.6	6.5
Homemaker	6.7	6.1	6.2
Student	5.8	1.7	1.6
Retired	17.9	32.3	33.5
Unable to work	6.9	13.9	27.4
**Health insurance**			
Insured	87.8	92.3	91.8
Uninsured	12.2	7.7	8.2
**Health status**			
Fair/poor health	17.5	31.5	51.5
Good or better health	82.5	68.5	48.5
**Disability status**			
Disabled^c^	20.3	37.6	61.3
Not disabled	79.7	62.4	38.7
**Smoking status**			
Current smoker	16.8	19.4	27.3
Nonsmoker	83.2	80.6	72.7

Abbreviation: MCC, multiple chronic conditions.

a MCC12: Any 2 or more of heart disease, stroke, asthma, arthritis, chronic obstructive pulmonary disease, high cholesterol, cognitive impairment, diabetes, depression, chronic kidney disease, cancer other than skin, hypertension.

b MCC5: Any 2 or more of cardiovascular disease, asthma, arthritis, chronic obstructive pulmonary disease, cognitive impairment.

c Limited in any way in any activities because of physical, mental, or emotional problems.

**Table 2:** Comparison of Adults Aged ≥65 Years With Those Aged <65 Years With Multiple Chronic Conditions (MCCs) on Selected Measures, Behavioral Risk Factor Surveillance System, 2015

Measure	Adults Reporting MCC12^a^			Adults Without MCC12^a^		
	<65 y (n = 99,549)	≥65 y (n = 100,458)	All Ages (N = 200,007)	<65 y (n = 154,290)	≥65 y (n = 35,412)	All Ages (N = 189,702)
	% (95% Confidence Interval)					
**Component of the MCC**						
Heart disease	10.7 (10.3–11.0)	22.9 (22.4–23.5)	15.1 (14.8–15.4)	0.3 (0.3–0.4)	1.5 (1.2–1.7)	0.4 (0.4–0.5)
Stroke	5.5 (5.2–5.7)	9.9 (9.5–10.2)	7.1 (6.8–7.3)	0.2 (0.1–0.2)	0.7 (0.5–1.0)	0.2 (0.2–0.3)
Any CVD	14.2 (13.8–14.6)	28.9 (28.3–29.5)	19.5 (19.2–19.8)	0.5 (0.4–0.6)	2.2 (1.8–2.5)	0.7 (0.6–0.7)
Arthritis	46.3 (45.7–46.9)	63.0 (62.3–63.6)	52.3 (51.9–52.8)	4.8 (4.6–5.0)	17.3 (16.4–18.2)	5.9 (5.7–6.0)
COPD	13.1 (12.7–13.5)	15.9 (15.4–16.3)	14.1 (13.8–14.4)	0.6 (0.5–0.7)	1.5 (1.2–1.7)	0.7 (0.6–0.8)
Asthma (current)	19.9 (19.5–20.4)^b^	10.6 (10.2–11.0)	16.6 (16.2–16.9)	3.7 (3.5–3.9)^b^	1.1 (0.9–1.3)	3.5 (3.3–3.6)
Cognitive impairment	26.7 (26.2–27.3)^b^	11.5 (11.1–11.9)	21.2 (20.8–21.6)	2.0 (1.9–2.2)^b^	0.8 (0.7–1.0)	1.9 (1.8–2.1)
Diabetes	20.9 (20.4–21.4)	29.1 (28.5–29.7)	23.8 (23.5–24.2)	1.1 (1.0–1.2)	3.2 (2.8–3.7)	1.3 (1.2–1.4)
Depression diagnosis	43.6 (43.0–44.2)^b^	19.1 (18.6–19.5)	34.7 (34.3–35.1)	5.9 (5.7–6.1)^b^	1.8 (1.5–2.0)	5.5 (5.3–5.7)
High blood pressure	59.1 (58.5–59.7)	75.7 (75.2–76.3)	65.1 (64.7–65.6)	8.3 (8.0–8.5)	18.9 (18.1–19.7)	9.2 (8.9–9.4)
Cancer other than skin	9.9 (9.6–10.3	22.1 (21.6–22.6)	14.3 (14.1–14.6)	1.1 (1.0–1.2)	5.2 (4.8–5.8)	1.5 (1.4–1.5)
Any of above	76.2 (75.7–76.8)	90.6 (90.2–90.9)	81.4 (81.0–81.8)	34.9 (34.4–35.3)	64.8 (63.8–65.8)	37.4 (37.0–37.8)
Kidney disease	5.3 (5.0–5.6)	7.6 (7.2–7.9)	6.1 (5.9–6.3)	0.3 (0.2–0.4)	0.3 (0.2–0.4)	0.3 (0.2–0.3)
High cholesterol	55.3 (54.7–55.9)	65.9 (65.3–66.5)	59.1 (58.7–59.6)	8.7 (8.4–8.9)	13.4 (12.7–14.1)	9.1 (8.8–9.3)
≥4 Chronic conditions	29.8 (29.3–30.3)	42.5 (41.8–43.1)	34.4 (34.0–34.8)	0	0	0
**Disability^c^ and related issues**						
Limited in activities	38.8 (38.2–39.3)^b^	35.7 (35.1–36.3)	37.7 (37.2–38.1)	7.1 (6.8–7.3)	11.8 (11.2–12.5)	7.5 (7.3–7.7)
Serious difficulty seeing	8.4 (8.1–8.8)^b^	7.2 (6.9–7.6)	8.0 (7.7–8.3)	1.7 (1.6–1.9)	2.9 (2.6–3.3)	1.8 (1.7–1.9)
Difficulty walking	25.4 (24.9–25.9)	31.7 (31.1–32.3)	27.7 (27.3–28.1)	2.7 (2.6–2.8)	9.0 (8.4–9.7)	3.2 (3.1–3.4)
Difficulty bathing	8.0 (7.7–8.4)^b^	6.7 (6.4–7.1)	7.6 (7.3–7.8)	0.6 (0.6–0.7)	1.3 (1.1–1.7)	0.7 (0.6–0.8)
Difficulty doing errands	15.0 (14.6–15.4)^b^	11.7 (11.2–12.1)	13.8 (13.5–14.1)	1.4 (1.3–1.5)	3.0 (2.6–3.4)	1.5 (1.4–1.6)
Unable to work	18.5 (18.1–19.0)^b^	5.9 (5.6–6.2)	13.9 (13.6–14.3)	1.6 (1.5–1.8)	1.7 (1.3–2.1)	1.6 (1.5–1.7)
**Health behaviors and risk factors**						
Obese (BMI ≥30 kg/m^2^)	43.4 (42.8–44.0)^b^	31.3 (30.7–31.9)	39.0 (38.5–39.4)	22.5 (22.1–22.9)^b^	16.0 (15.3–16.9)	21.9 (21.6–22.3)
Current smoking	25.5 (24.9–26.0)^b^	8.8 (8.5–9.2)	19.5 (19.1–19.8)	15.0 (14.6–15.3)^b^	8.2 (7.7–8.8)	14.4 (14.1–14.7)
No leisure time activity	31.7 (31.1–32.3)	33.5 (32.9–34.1)	32.4 (31.9–32.8)	20.5 (20.1–20.9)	22.0 (21.1–22.9)	20.6 (20.3–21.0)
**Health access measures**						
No health care coverage	11.2 (10.8–11.7)^b^	1.6 (1.4–1.8)	7.7 (7.5–8.1)	15.8 (15.5–16.2)^b^	2.3 (2.0–2.6)	14.7 (14.3–15.0)
No personal physician	13.6 (13.1–14.1)^b^	3.3 (3.1–3.5)	9.9 (9.6–10.2)	30.2 (29.8–30.7)^b^	9.9 (9.2–10.6)	28.5 (28.1–28.9)
Cost barrier to care^d^	20.8 (20.3–21.3)^b^	5.5 (5.1–5.8)	15.3 (14.9–15.6)	11.7 (11.4–12.0)^b^	3.3 (2.8–3.9)	11.0 (10.7–11.3)
Check-up in 2 years	87.6 (87.1–88.0)^b^	95.9 (95.6–96.2)	90.6 (90.3–90.9)	77.5 (77.1–77.9)^b^	89.1 (88.4–89.7)	78.5 (78.1–78.9)
**Health-related quality of life**						
Fair or poor general health	32.6 (32.0–33.2)^b^	29.7 (29.1–30.3)	31.6 (31.1–32.0)	6.6 (6.4–6.9)	7.7 (7.1–8.4)	6.7 (6.5–7.0)
Frequent mental distress^e^	23.9 (23.3–24.4)^b^	8.6 (8.2–9.0)	18.4 (18.0–18.7)	6.3 (6.0–6.5)^b^	2.6 (2.2–3.0)	5.9 (5.7–6.2)
Frequent physical distress^e^	23.3 (22.8–23.8)^b^	20.2 (19.6–20.7)	22.2 (21.8–22.5)	4.3 (4.1–4.5)	5.7 (5.2–6.2)	4.5 (4.3–4.6)
Frequent activity limitation^e^	18.1 (17.6–18.5)^b^	11.6 (11.2–12.0)	15.7 (15.4–16.1)	2.3 (2.1–2.4)	2.6 (2.3–3.0)	2.3 (2.2–2.4)

Abbreviations: BMI, body mass index; CVD, cardiovascular disease.

a MCC12: Any 2 or more of heart disease, stroke, asthma, arthritis, chronic obstructive pulmonary disease, high cholesterol, cognitive impairment, diabetes, depression, chronic kidney disease, cancer other than skin, hypertension.

b Result for adults <65 y is less desirable than for those aged ≥65 y (*P* < .05 by Pearson χ^2^ test).

c Limited in any way in any activities because of physical, mental, or emotional problems.

d Needed to see a doctor in the past year but could not because of cost.

e Frequent: ≥14 days out of the past 30 days.

Logistic regression analysis for the 10 measures that showed similar results for adults with and without MCC12 resulted in adjusted odds ratios (AORs) comparing younger with older adults that ranged from 1.49 (95% confidence interval [CI], 1.36–1.63) for FMD to 4.79 (95% CI, 4.01–5.71) for being uninsured (Table 3). For the 8 outcomes that showed significant age differences among adults with MCC12 but not among those without MCC12, only the AORs for disability, fair or poor general health, FAL, and being unable to work that compared respondents aged 18 to 64 years versus those aged 65 years or older among adults with MCC12 were higher than 1.00 (Table 3). AORs comparing non-elderly versus elderly adults with MCC12 for difficulty seeing and FPD were not significantly different from 1.00, but AORs for difficulty dressing and running errands were less than 1.00 indicating adults aged 65 or older with MCC12 were more likely to report those outcomes. Results for MCC5 were similar to those for MCC12 (Appendix).

**Table 3:** Logistic Regression Analysis of Selected Measures, Adults With MCC12^a^, Adjusted for All Measures, Behavioral Risk Factor Surveillance System, 2015

Characteristic	Disability	Fair/Poor Health	FAL^b^	FMD^c^
Measure	Adjusted Odds Ratio (95% Confidence Interval)			
**Sex**				
Male	1 [Reference]			
Female	0.86 (0.82–0.90)	0.93 (0.89–0.99)	0.98 (0.91–1.05)	1.15 (1.07–1.23)
**Age, y**				
≥65	1 [Reference]			
18–64^d^	1.10 (1.03–1.17)	1.11 (1.03–1.18)	1.15 (1.05–1.27)	1.49 (1.36–1.63)
**Race/ethnicity**				
White, non-Hispanic	1 [Reference]			
Black, non-Hispanic	0.58 (0.53–0.63)	1.33 (1.23–1.45)	0.94 (0.84–1.06)	0.95 (0.85–1.06)
Hispanic	0.58 (0.52–0.65)	2.51 (2.27–2.78)	1.09 (0.94–1.27)	0.99 (0.86–1.13)
American Indian/Alaska Native	0.85 (0.69–1.05)	1.41 (1.14–1.75)	1.03 (0.78–1.36)	1.16 (0.94–1.44)
Other	0.83 (0.70–0.97)	1.30 (1.10–1.54)	1.36 (1.12–1.65)	1.05 (0.88–1.25)
**Education level**				
College graduate	1 [Reference]			
Some college	0.93 (0.88–0.99)	1.23 (1.15–1.31)	1.04 (0.95–1.14)	1.15 (1.06–1.25)
High school graduate	0.66 (0.62–0.70)	1.57 (1.47–1.67)	1.00 (0.91–1.10)	1.22 (1.12–1.33)
<High school	0.52 (0.47–0.57)	2.31 (2.10–2.54)	1.00 (0.88–1.15)	1.10 (0.97–1.24)
**Annual household income, $**				
≥75,000	1 [Reference]			
50,000–74,999	0.98 (0.91–1.06)	1.25 (1.14–1.38)	1.13 (0.97–1.30)	1.17 (1.04–1.33)
25,000–49,999	1.10 (1.03–1.18)	1.48 (1.36–1.61)	1.08 (0.96–1.22)	1.13 (1.01–1.26)
15,000–24,999	1.07 (0.99–1.17)	1.71 (1.56–1.88)	1.07 (0.94–1.22)	1.20 (1.07–1.34)
<15,000	1.18 (1.06–1.30)	1.95 (1.75–2.17)	1.13 (0.98–1.29)	1.27 (1.12–1.44)
Unknown	0.92 (0.84–1.00)	1.73 (1.57–1.91)	1.07 (0.93–1.22)	1.13 (1.01–1.28)
**Employment status**				
Employed/self-employed	1 [Reference]			
Out of work	2.04 (1.83–2.28)	1.15 (1.01–1.30)	2.62 (2.26–3.03)	1.33 (1.17–1.51)
Homemaker	1.52 (1.37–1.69)	1.31 (1.16–1.47)	1.76 (1.49–2.07)	0.93 (0.81–1.08)
Student	1.49 (1.22–1.81)	0.85 (0.68–1.07)	1.34 (0.93–1.93)	1.13 (0.89–1.43)
Retired	1.88 (1.76–2.02)	1.11 (1.03–1.19)	2.01 (1.79–2.26)	0.82 (0.74–0.90)
Unable to work	8.92 (8.10–9.82)	2.20 (2.01–2.40)	3.97 (3.55–4.45)	1.10 (1.00–1.21)
**Obesity status**				
Not obese	1 [Reference]			
Obese (BMI ≥30 kg/m^2^)	1.28 (1.22–1.35)	1.24 (1.18–1.31)	0.97 (0.90–1.04)	0.97 (0.91–1.04)
**Smoking status**				
Nonsmoker	1 [Reference]			
Current smoker	1.04 (0.98–1.12)	1.02 (0.95–1.09)	0.99 (0.91–1.09)	1.38 (1.27–1.50)
**FMD^c^**				
No FMD	1 [Reference]			
FMD	1.51 (1.40–1.63)	2.03 (1.89–2.18)	5.14 (4.73–5.59)	—
**Barrier to health care^e^**				
No barrier	1 [Reference]			
Cost barrier	1.31 (1.20–1.42)	1.48 (1.37–1.60)	1.36 (1.24–1.50)	1.47 (1.36–1.60)
**Disability^f^**				
No disability	1 [Reference]			
Disability	—	2.91 (2.76–3.08)	3.79 (3.50–4.11)	1.53 (1.42–1.65)
**Health status**				
Good or better health	1 [Reference]			
Fair/poor health	2.88 (2.73–3.05)	—	3.01 (2.79–3.24)	2.10 (1.96–2.26)
**Chronic condition**				
None	1 [Reference]			
Diabetes	1.06 (1.00–1.12)	1.98 (1.86–2.10)	1.08 (1.00–1.17)	0.89 (0.83–0.96)
Depression	1.52 (1.44–1.61)	1.03 (0.97–1.10)	1.10 (1.01–1.19)	4.23 (3.96–4.53)
Hypertension	1.02 (0.97–1.07)	1.36 (1.28–1.44)	1.04 (0.96–1.13)	1.03 (0.96–1.10)
High cholesterol	0.98 (0.94–1.03)	1.07 (1.01–1.13)	0.97 (0.90–1.04)	0.98 (0.92–1.04)
Asthma	1.20 (1.11–1.28)	1.15 (1.06–1.23)	1.09 (0.99–1.20)	1.26 (1.16–1.37)
Cardiovascular disease	1.41 (1.33–1.49)	1.95 (1.83–2.07)	1.06 (0.98–1.15)	0.99 (0.92–1.08)
Cognitive impairment	2.68 (2.50–2.88)	1.30 (1.22–1.40)	1.46 (1.34–1.59)	3.18 (2.96–3.41)
Arthritis	2.66 (2.53–2.79)	1.35 (1.28–1.42)	1.26 (1.17–1.36)	0.92 (0.86–0.99)
COPD	1.61 (1.50–1.72)	1.96 (1.83–2.10)	1.26 (1.16–1.38)	1.04 (0.96–1.14)
Cancer other than skin	1.19 (1.12–1.26)	1.63 (1.52–1.74)	1.12 (1.02–1.23)	0.97 (0.89–1.05)

Abbreviations: — , not applicable; BMI, body mass index; COPD, chronic obstructive pulmonary disease; FAL, frequent activity limitations; FMD, frequent mental distress; MCC, multiple chronic conditions.

a MCC12: Any 2 or more of heart disease, stroke, asthma, arthritis, chronic obstructive pulmonary disease, high cholesterol, cognitive impairment, diabetes, depression, chronic kidney disease, cancer other than skin, hypertension. N = 177,511 for those with FAL, and N = 179,201 for all others.

b FAL is ≥14 days of activity limitation in the past 30 days.

c FMD is ≥14 days poor mental health in the past 30 days.

d All *P* value for age <.01.

e Needed to see a doctor in the past year but could not because of cost.

f Limited in any way in any activities because of physical, mental, or emotional problems.

## Discussion

Among adults with 2 MCCs there were significant differences by age group in 18 measures, indicating that adults younger than 65 were worse off than adults aged 65 or older. Results were similar whether diabetes, depression, hypertension, high cholesterol (which could also be risk factors) were included in the MCC (MCC12) or not (MCC5). For 14 of those measures among adults with MCC12 (3 component chronic conditions, 2 risk factors, 4 access to health care measures, 2 disability/employment measures, and 3 quality of life measures) age group differences remained after adjustment for demographic and health measures. These included 10 measures that had similar results for adults with and without MCC12 and 4 measures (disability, being unable to work, fair or poor health and FAL) that showed significant age differences only among adults with the MCC. 

Some of these age differences for adults with MCCs can be easily explained by similar differences among adults without the MCC, but the results could still be important. For example, most uninsured adults are younger than 65 years, and younger adults with MCCs were more likely than older adults to report a cost barrier to their health care in the past year. Younger adults with MCCs were also less likely to report a recent routine check-up than adults aged 65 or older with MCCs. These are all important findings in light of the continuing need to manage and treat existing chronic conditions and diagnose incident ones. This finding appears to be somewhat inconsistent with results showing that medical care expenditures for chronic conditions for adults aged 18 to 44, 45 to 64, and 65 or older with MCCs were similar at approximately $9,000 for those with 4 or more chronic conditions and approximately $4,000 for those with 2 or 3 (6). However, those data included only expenditures for those who received care for their chronic conditions, and younger adults with MCCs were less likely than older adults with similar numbers of MCCs to be treated (6). Thus, some people (eg, the uninsured, those reporting a cost barrier to care in this study) may have been unable to get needed treatment for their chronic conditions.

Quality of life measures have been studied before using BRFSS data (19), and results indicated that people with 3 or more chronic conditions and those with CVD or diabetes were more likely to report poorer quality of life than those with fewer or different chronic conditions; however, this analysis did not compare age groups. Although not directly compared, adults with MCC12 in our study appeared more likely to report poorer quality of life than adults without MCC12. Some of our study findings may result from age differences in the composition of the MCCs (eg, more depression and cognitive impairment among younger adults), which might have a greater effect on mental health compared with other chronic conditions. In particular, higher rates of depression among younger adults may be a crucial factor, because depression is the leading cause of disability worldwide (20). That fact would not fully explain results, however, because only MCC12 included depression whereas MCC5 did not, and rates of FMD were higher for both age groups among adults with MCC5 compared with adults with MCC12. Differences in reported quality of life could also result from different contexts, because younger adults were much more likely to be employed than older adults. For example, interpretation of activity limitations may depend on age, employment status, or both. However, even when controlling for measures including employment status and depression, these age differences remained for 14 measures representing a range of outcomes. These results may also reflect the direct or indirect impact of other factors such as smoking or obesity that are also higher among younger adults and may affect health and disability status. Whatever the cause of the differences, results highlight the current impact of MCCs on younger adults.

The higher rates of asthma, depression, and cognitive impairment among younger adults with MCCs were not totally unexpected. Age-related differences in MCCs using hospital discharge data indicated, for example, that among adults 18 to 44 years, the dyad of depression and substance abuse was most common (21). Our results, which lack information on substance abuse, are consistent with the earlier finding, by showing that compared with elderly adults, depression was more common among working age adults with or without MCCs. Along with depression, risk factors of obesity and smoking were also higher among younger adults with and without MCCs. Rates of hypertension, high cholesterol, and diabetes were all lower among younger adults with MCCs, but because these risk factors can also be risk factors for other chronic conditions (8–10), they may still be important. The presence of these well-recognized risk factors suggests that interventions are needed to target nonelderly adults with MCCs to reduce their risk of developing additional chronic conditions as they age.

The inclusion of cognitive impairment in the measures is consistent with lists of chronic conditions that include dementia, autism, and schizophrenia (14), or addiction, mental illnesses, cognitive impairment, and developmental disabilities (15), any of which can complicate management of co-occurring chronic conditions. A somewhat unexpected finding was the high rate of cognitive impairment among younger adults with MCCs. This rate could result from lower rates of other chronic conditions or factors such as lack of sleep, side effects of medication, or use of illicit drugs and may not be associated with future risk of dementia (9,10). Whatever the cause, being cognitively impaired may affect someone’s ability to self-manage other chronic conditions (22).

Findings from this study add to existing information on MCCs among all adults. First, results showed that despite the fact that rates of MCCs increase with age, most adults with MCCs by these definitions were younger than age 65. Second, younger adults with these MCCs were more likely to report 14 measures, including disability and poorer quality of life, than were older adults with the same MCCs. Some age differences found in this study for adults with MCCs can be explained by similar differences between the age groups among adults without the MCC, but they may still be important. Although poorer access to health care was common among all adults younger than 65, this factor could affect management and treatment of existing chronic conditions and identification of new ones. Younger adults were also more likely than older adults to report risk factors that can increase risk of developing additional chronic conditions and thus higher rates of MCCs in the future.

Limitations of this study include the use of self-reported data and the sample being limited to noninstitutionalized adults, which likely excluded many older adults with MCCs in long-term care. Some respondents may have been unaware of a diagnosis, in which case MCCs may have been underreported. Autism, hepatitis, HIV/AIDS, osteoporosis, psychotic disorders, addiction and developmental disabilities that are not measured on the BRFSS were excluded. A strength of the study was its use of data from a large population-based survey of US adults, which makes results generalizable to all states.

Findings from this study show the extent of the burden of MCCs on nonelderly adults who represent more than 60% of all adults with MCCs. Any strategies to manage and treat chronic conditions may be affected by the poorer access to health care reported by younger adults. Interventions to address the risk factors of hypertension, high cholesterol, diabetes, depression, obesity, and smoking are needed to reduce or delay the development of additional chronic conditions. Successful interventions can reduce the burden of chronic conditions and reduce present and future health care costs from MCCs.

## Appendix Supplemental Tables

**Table:** Table S-1. Comparison of Adults Aged ≥65 Years With Those Aged <65 Years Reporting MCC5^a^ on Selected Measures, Behavioral Risk Factor Surveillance System (N = ~69,000)

Component of the MCC	<65 Years	≥65 Years	All Ages
	% (95% Confidence Interval)		
**Any cardiovascular disease**	32.2 (31.3–33.1)	60.1 (59.1–61.1)	42.9 (42.2–43.7)
**Arthritis**	77.7 (76.8–78.6)	87.3 (86.6–88.0)	81.4 (80.8–82.0)
**COPD**	36.1 (35.1–37.0)	39.2 (38.2–40.1)	37.3 (36.6–38.0)
**Asthma (current)**	44.1 (43.0–45.1)^b^	26.5 (25.6–27.4)	37.3 (36.5–38.0)
**Cognitive impairment**	57.2 (56.2–58.2)^b^	28.6 (27.6–29.6)	46.2 (45.4–46.9)
**Disability and related issues**			
Limited in activities only^c^	65.8 (64.8–66.8)^b^	54.2 (53.2–55.3)	61.4 (60.6–62.1)
Employed	35.2 (34.2–36.2)	8.4 (7.9–9.0)	24.9 (24.2–25.5)
Unable to work	37.8 (36.8–38.8)^b^	11.0 (10.3–11.7)	27.5 (26.8–28.1)
**Health behaviors and risk factors**			
Obese (BMI ≥30 kg/m^2^)	45.4 (44.4–46.4)^b^	36.1 (35.1–37.1)	41.8 (41.0–42.5)
Current smoking	37.2 (36.2–38.2)^b^	11.9 (11.3–12.6)	27.4 (26.8–28.1)
No leisure time activity	39.7 (38.6–40.7)	41.5 (40.5–42.5)	40.4 (39.6–41.1)
**Health access measures**			
No health care coverage	12.1 (11.4–12.9)^b^	2.0 (1.6–2.5)	8.2 (7.7–8.7)
No personal doctor or doctors	13.2 (12.4–14.0)^b^	3.5 (3.2–3.9)	9.5 (9.0–10.0)
Unable to see doctor^d^	29.4 (28.4–30.3)^b^	8.5 (7.8–9.2)	21.3 (20.7–22.0)
Check-up in 2 years	86.7 (85.9–87.4)^b^	95.2 (94.7–95.7)	90.0 (89.5–90.4)
**Health-related quality of life**			
Fair or poor health status	54.1 (53.1–55.1)^b^	47.3 (46.3–48.3)	51.5 (50.7–52.2)
Frequent mental distress^e^	39.9 (38.9–40.9)^b^	15.2 (14.4–16.0)	30.4 (29.7–31.1)
Frequent physical distress^e^	43.2 (42.2–44.2)^b^	33.8 (32.8–34.8)	39.6 (38.9–40.3)
Frequent activity limitation^e^	36.3 (35.3–37.3)^b^	20.9 (20.0–21.8)	30.4 (29.7–31.1)

Abbreviations: BMI, body mass index; COPD, chronic obstructive pulmonary disease; MCC, multiple chronic conditions.

^a^ MCC5: Any 2 or more of cardiovascular disease, asthma, arthritis, chronic obstructive pulmonary disease, cognitive impairment.

^b^ Result for adults <65 y is less desirable than for those aged ≥65 y (*P* < .05 by Pearson χ^2^ test ).

^c^ Limited in any way in any activities because of physical, mental, or emotional problems.

^d^ Needed to see a doctor in the past year but could not due to cost.

^e^ Frequent means ≥14 days of the past 30 days.

**Table:** Table S-2. Adjusted Odds Ratios and 95% Confidence Intervals for Selected Measures, Adults With MCC5^a^, Adjusted for All Measures Listed, Behavioral Risk Factor Surveillance System, 2015 (N = ~ 59,000)

Measure	Disability	Fair/Poor Health	FAL^b^	FMD^c^
	Adjusted Odds Ratio (95% Confidence Interval)			
**Sex**				
Men	1 [Reference]			
Women	0.81 (0.75–0.88)	0.98 (0.91–1.06)	1.03 (0.93–1.13)	1.08 (0.98–1.19)
**Age, y^d^**				
≥65	1 [Reference]			
18–64	1.17 (1.05–1.30)	1.13 (1.02–1.25)	1.14 (1.01–1.30)	1.48 (1.31–1.68)
**Race/ethnicity**				
White, non-Hispanic	1 [Reference]			
Black, non-Hispanic	0.58 (0.51–0.67)	1.12 (0.98–1.27)	0.89 (0.76–1.03)	0.86 (0.75–1.00)
Hispanic	0.58 (0.48–0.70)	1.98 (1.64–2.39)	1.05 (0.87–1.27)	0.87 (0.72–1.06)
American Indian/Alaska Native	0.74 (0.55–1.01)	1.26 (0.90–1.77)	1.06 (0.75–1.51)	1.17 (0.90–1.53)
Other	0.96 (0.75–1.23)	1.08 (0.85–1.37)	1.56 (1.18–2.05)	1.14 (0.88–1.47)
**Education level**				
College graduate	1 [Reference]			
Some college	0.90 (0.81–0.99)	1.24 (1.12–1.36)	1.01 (0.89–1.14)	1.03 (0.91–1.16)
High school graduate	0.61 (0.55–0.68)	1.43 (1.30–1.58)	0.98 (0.86–1.11)	1.06 (0.94–1.20)
< High school graduate	0.48 (0.42–0.56)	2.01 (1.75–2.30)	0.94 (0.79–1.11)	1.15 (0.98–1.35)
**Annual household income, $**				
≥75,000	1 [Reference]			
50,000–74,999	1.00 (0.86–1.15)	1.15 (0.99–1.34)	0.99 (0.81–1.21)	1.20 (0.98–1.47)
25,000–49,999	1.06 (0.93–1.20)	1.29 (1.13–1.48)	0.94 (0.79–1.12)	1.15 (0.96–1.37)
15,000–24,999	1.06 (0.92–1.22)	1.46 (1.26–1.69)	0.96 (0.80–1.15)	1.23 (1.02–1.48)
<15,000	1.17 (1.00–1.37)	1.71 (1.45–2.01)	1.03 (0.85–1.25)	1.34 (1.10–1.62)
Unknown	0.91 (0.79–1.05)	1.49 (1.29–1.74)	1.00 (0.82–1.23)	1.23 (1.01–1.51)
**Employment status**				
Employed/self-employed	1 [Reference]			
Out of work	2.21 (1.83–2.68)	1.17 (0.97–1.40)	2.88 (2.33–3.46)	1.44 (1.17–1.78)
Homemaker	1.70 (1.43–2.02)	1.24 (1.04–1.49)	1.93 (1.53–2.44)	0.89 (0.72–1.12)
Student	1.35 (0.95–1.92)	0.71 (0.49–1.04)	1.48 (0.74–3.00)	1.09 (0.73–1.63)
Retired	1.82 (1.62–2.04)	1.24 (1.11–1.39)	2.02 (1.72–2.37)	0.83 (0.72–0.97)
Unable to work	6.51 (5.66–7.48)	2.42 (2.13–2.75)	3.72 (3.20–4.33)	.09 (0.96–1.25)
**Obesity (BMI ≥30 kg/m^2^)**				
Not obese	1 [Reference]			
Obese	1.25 (1.15–1.36)	1.18 (1.09–1.28)	1.03 (0.94–1.13)	0.99 (0.90–1.09)
**Smoking status**				
Nonsmoker	1 [Reference]			
Current smoker	0.98 (0.89–1.09)	1.00 (0.91–1.10)	0.95 (0.85–1.05)	1.30 (1.17–1.45)
**FMD**				
No FMD	1 [Reference]			
FMD	1.40 (1.22–1.52)	1.89 (1.71–2.09)	4.41 (4.00–4.90)	—
**Barrier to health care**				
None	1 [Reference]			
Cost barrier	1.31 (1.17–1.47)	1.33 (1.20–1.48)	1.40 (1.26–1.61)	1.48 (1.32–1.65)
**Disability**				
No disability	1 [Reference]			
Disability	—	2.72 (2.51–2.95)	3.20 (2.83–3.58)	1.40 (1.21–1.52)
**Health status**				
Good or better health	1 [Reference]			
Fair/poor health	2.73 (2.52–2.96)	—	2.96 (2.68–3.27)	2.00 (1.77–2.15)
**Chronic conditions (CCs)**				
Each vs not the CC	1 [Reference]			
Diabetes	1.06 (0.97–1.16)	1.71 (1.56–1.86)	1.05 (0.95–1.16)	0.88 (0.79–0.98)
Depression	1.52 (1.39–1.66)	1.03 (0.95–1.13)	1.12 (1.01–1.24)	4.18 (3.79–4.60)
Hypertension	1.04 (0.95–1.13)	1.34 (1.24–1.46)	1.02 (0.92–1.13)	1.06 (0.96–1.17)
High cholesterol	1.10 (1.02–1.19)	1.02 (0.95–1.11)	1.09 (1.00–1.20)	1.01 (0.92–1.11)
Asthma	1.15 (1.05–1.26)	1.04 (0.95–1.14)	1.08 (0.98–1.20)	1.19 (1.08–1.32)
Cardiovascular disease	1.27 (1.15–1.40)	1.75 (1.61–1.91)	0.99 (0.89–1.09)	0.97 (0.87–1.07)
Cognitive impairment	2.33 (2.10–2.58)	1.29 (1.17–1.42)	1.43 (1.28–1.60)	2.95 (2.66–3.26)
Arthritis	1.94 (1.73–2.18)	1.30 (1.16–1.46)	1.28 (1.11–1.48)	0.95 (0.84–1.07)
COPD	1.44 (1.32–1.58)	1.76 (1.62–1.91)	1.24 (1.12–1.38)	1.00 (0.90–1.10)
Cancer not skin	1.23 (1.11–1.35)	1.44 (1.30–1.59)	1.07 (0.95–1.22)	1.01 (0.90–1.13)

Abbreviations: —, not applicable; BMI, body mass index; CC, chronic condition; COPD: chronic obstructive pulmonary disease; FAL, frequent activity limitation; FMD, frequent mental distress.

^a^ MCC5: Any 2 or more of cardiovascular disease, asthma, arthritis, COPD, cognitive impairment.

^b^ FAL refers to ≥14 days of activity limitation in past 30 days.

^c^ FMD refers to ≥14 days of poor mental health in past 30 days.

^d^ All *P* values for age <.05.
